# Development of Novel PET-PAN Electrospun Nanocomposite Membrane Embedded with Layered Double Hydroxides Hybrid for Efficient Wastewater Treatment

**DOI:** 10.3390/polym15224388

**Published:** 2023-11-12

**Authors:** Abdul Majeed Pirzada, Imran Ali, Nabi Bakhsh Mallah, Ghulamullah Maitlo

**Affiliations:** 1 Department of Environmental Sciences, Sindh Madressatul Islam University, Karachi 74000, Pakistan; 2 Faculty of Engineering, Science and Technology, Hamdard University, Karachi 75210, Pakistan; nabi.bakhsh@hamdard.edu.pk; 3 Department of Chemical Engineering, Dawood University of Engineering and Technology, Karachi 74800, Pakistan; ghulam.maitlo@duet.edu.pk

**Keywords:** layered double hydroxides, electrospinning, nanocomposite membrane, adsorption, wastewater treatment

## Abstract

Layered double hydroxides (LDHs) with their unique structural chemistry create opportunities to be modified with polymers, making different nanocomposites. In the current research, a novel PET-PAN embedded with Mg-AI-LDH-PVA nanocomposite membrane was fabricated through electrospinning. SEM, EDX, FTIR, XRD, and AFM were carried out to investigate the structure and morphology of the nanocomposite membrane. The characterization of the optimized nanocomposite membrane showed a beadless, smooth structure with a nanofiber diameter of 695 nm. The water contact angle and tensile strength were 16° and 1.4 Mpa, respectively, showing an increase in the hydrophilicity and stability of the nanocomposite membrane by the addition of Mg-Al-LDH-PVA. To evaluate the adsorption performance of the nanocomposite membrane, operating parameters were achieved for Cr(VI) and methyl orange at pH 2.0 and pH 4.0, respectively, including contact time, adsorbate dose, and pollutant concentration. The adsorption data of the nanocomposite membrane showed the removal of 68% and 80% for Cr(VI) and methyl orange, respectively. The process of adsorption followed a Langmuir isotherm model that fit well and pseudo-2nd order kinetics with R^2^ values of 0.97 and 0.99, respectively. The recycling results showed the membrane’s stability for up to five cycles. The developed membrane can be used for efficient removal of pollutants from wastewater.

## 1. Introduction

The generation of wastewater from different industries badly affects the quality of ground and surface water, which in turn shows effects on different ecosystems in the environment and human health [1,2]. Therefore, the management and treatment of wastewater is a core issue worldwide. Recently, heavy metals and dye pollutants are continuously receiving the increased attention of researchers because of their adverse impact on the environment and human beings as well. Therefore, there is a need to purify dye contaminants and heavy metallic ions before their release into the environment [3]. Various kinds of methods have been used in order to treat dyes and heavy metallic ions from wastewater, for example, coagulation–flocculation, chemical precipitation, ion exchange, photocatalytic degradation, and membrane filtration [3,4,5]. However, these processes/methods have many disadvantages, such as consumption of high energy, they are expensive, and they have toxic byproducts. Therefore, adsorption has been considered as one of the promising methods due to its easy operation, it is economical, it can be recycled easily, and it is very efficient compared to conventional techniques [6,7].

The technique of electrospinning nanofiber membranes is known for its greater capability of treating industrial wastewater [8,9,10,11,12] because of its adjustable structure, higher level of efficiency, high porosity, greater surface area, simplicity, improved level of scalability, chemical reactivity, and highly efficient fabrication process with low cost [13,14,15,16,17,18]. At present, most researchers have used various inorganic, organic, inorganic/organic composite blends of polymers, and polymers containing nanoparticles in order to fabricate electrospun nanofiber membranes for treating heavy metallic ions and dyes [16,19,20,21,22].

Inexpensive polyacrylonitrile (PAN) contains a nitrile group (CN) that can be easily reacted with different types of polymers for producing electrospun nanofibers through the electrospinning technique. PAN is very efficient with a high recycling rate and high adsorption capability for pollutants [23]. Furthermore, PAN has a good level of thermal stability and chemical resistance along with a good level of mechanical properties [24,25]. Polyethylene terephthalate (PET) is a thermoplastic polymer with carboxyl and hydroxyl end groups that is extensively used in the packaging of drinking water and soft drink bottles. After usage, PET bottles are discarded. Therefore, the recycling of PET polymers has received huge attention in the last decades as well as for its application in pollutant removal [26]. This recycling potential of PET helps to reduce the burden of pollution on the environment. PET has good morphology, it is very economical and easily available, and it has high tensile strength [27,28].

Layered double hydroxide (LDH) is also called anionic clay; its shape consists of two-dimensional nanostructures, just like the brucite structure. LDH contains positive charge-based metallic hydroxyl layers with intercalated exchangeable anions for charge neutrality and water molecules. The selection of LDH is advantageous because its synthesis process is very simple, it has good stability, its structure can be adjustable, it has a large surface area and uniform distribution of positive charges over the surface, and it has the ability to be synthesized in different types of composites/hybrids with interplanar spacing. Because of these reasons, LDH has a great ability to play the role of adsorbent in order to treat wastewater. Poly(vinyl alcohol) (PVA) is a polymer that can be easily dissolved in water and has multiple active hydroxide groups. It is recognized with various attributes, such as a good level of mechanical strength, non-toxicity, low cost, excellent level of biocompatibility, and good electrospinnability [29].

Previously, different variants of polymer combinations have been used for the purpose of creating electrospun nanofiber membranes for treating wastewater. Peng Xu et al. (2022) prepared PPAN-PEI through the use of electrospinning for removing methyl orange [30]. In other research, Reza Khalili et al. (2022) first prepared PAN/graphene oxide nanofibers via the technique of electrospinning and, following that, the bimetallic nickel iron layer double hydroxide NiFe LDH/PAN/GO was prepared by the hydrothermal method for removing mixtures of dyes and metals [31].

Other research pointed out that Esra Altay Ozturk et al. (2022) prepared poly (L-lactic acid)/poly(ethylene oxide)-based composite electrospun fibers accompanied with magnesium-aluminum layered double hydroxide nanoparticles [21]. In another study, Abdullah M. Aldawsari et al. (2021) prepared a nanocomposite by using layered double hydroxide along with activated carbon Mg/AI-NO_3_-LDH-AC via the hydrothermal treatment method. The fabricated nanocomposite showed an extra ordinary level of attraction for dyes [32]. Gaofeng Zheng et al. (2021) synthesized PEO nanofibers using a TiO_2_ suspension via the electrospinning process. The functional nanoparticles distributed on the electrospun nanofibrous membrane could improve the surface functional performance of the membrane [33].

According to the literature, no researcher has yet synthesized the combination of a PET-PAN electrospun nanocomposite membrane embedded with Mg-Al-LDH-PVA for treating wastewater. Therefore, the aim of this research was to efficiently synthesize a novel PET-PAN embedded with Mg-Al-LDH-PVA nanocomposite membrane using the electrospinning technique and its subsequent characterization with FTIR, AFM, SEM, XRD, EDX, tensile strength, and water contact angle. The optimization of the PET-PAN embedded with Mg-Al-LDH-PVA electrospun nanocomposite membrane was carried out for the removal of hexavalent chromium (Cr(VI)) and methyl orange (MO). For the developed membrane, the adsorption behaviors of Cr(VI) and MO were studied with different parameters. Various operating parameters, such as adsorbate concentration, pH, adsorbent dosage, and time, that can affect adsorption were tested. In addition, to check the membrane efficiency, kinetics, isotherm, and reusability tests were conducted.

## 2. Materials and Methods

### 2.1. Materials

In the current study, homopolymers were used for the preparation of the electrospun membrane. Polyethylene terephthalate (PET) post-consumer bottles were used without further purification. Polyacrylonitrile (PAN) in white powder form with an average molecular weight (Mw) = 150,000 was purchased from Sigma-Aldrich Corporation, Burlington, MA, USA. Similarly, this research also used other polymers and chemicals, namely polyvinyl alcohol (PVA) with a molecular weight (Mw) = 9000–10,000 (80% hydrolyzed), aluminum nitrate nonahydrate (Al(NO_3_)_3_·9H_2_O, ≥98%), magnesium nitrate hexahydrate (Mg(NO_3_)_2_·6H_2_O, 99%), sodium carbonate powder (Na_2_CO_3_, ≥99.5%), sodium hydroxide pellets (NaOH), hydrochloric acid (HCI, 37%), 1,5-diphenyl carbazide (DPC), sulfuric acid, and acetone, which were obtained from Sigma-Aldrich corporation, Burlington, MA, USA. N,N-Dimethylformamide (DMF), dichloromethane (DCM), trifluoroacetic acid (TFA), potassium dichromate (K_2_Cr_2_O_7_), and methyl orange (MO) were obtained from Dae-Jung company, Busan, Republic of Korea. Besides that, this research also used ultra-pure water and analytical grade chemicals for all experiments.

### 2.2. Synthesis of Mg-Al-LDH Nanoparticles

The coprecipitation method was used for the synthesis of Mg-A1-LDH [34,35]. For the synthesis of Mg-Al-LDH nanoparticles, initially Mg (NO_3_)_2_·6H_2_O and Al (NO_3_)_3_·9H_2_O were employed at constant pH (9.0–9.5) and a ratio of 3:1 (Mg: AI) as precursors. Typically, 50 mL of mixed aqueous solution of Mg (NO_3_)_2_·6H_2_O (0.075 mol) and Al (NO_3_)_3_·9H_2_O (0.025 mol) was mixed with 50 mL of Na_2_CO_3_ (0.05 mol) and NaOH (1 mol) aqueous solution, added dropwise with vigorous stirring. The pH was maintained in the range of 9.0 to 9.5 with the help of NaOH (1 mol) solution. The slurry was then aged for a duration of 12 h and temperature of 65 °C using a magnetic stirrer (as shown in the Supplementary Data, Figure S1). The solid thus obtained was then centrifuged, washed three times with deionized water, and finally dried in an oven for a duration of 12 h at 70 °C.

### 2.3. Preparation of Polymer Solutions for Electrospinning

For the preparation of PET polymer solution for electrospinning, initially the waste PET bottles were collected, then cut into square-shaped pieces 1 × 1 cm^2^ in size and subsequently cleaned and rinsed with deionized water for three times. In order to remove impurities, the pieces of PET bottle were heated at 40 °C for 30 min in ethanol solution. Then, for preparing the required 5 wt.% homogeneous solution, the pieces of PET bottle were dissolved in a mixture of DCM and TFA at a ratio of 3:1 [36]. Subsequently, the resulting solution was stirred for 4 h at room temperature. Meanwhile, the 8 wt.% polyacrylonitrile (PAN) solution was dissolved in DMF by continuous stirring at ambient temperature for 12 h. Similarly, for preparing the 8 wt.% PVA solution, the polymers were dissolved in double-distilled water for 5 h at a temperature of 80 °C with continuous stirring. Then, different quantities of Mg-Al-LDH nanoparticles, i.e., 0.08, 0.12, and 0.16 g, were added to the PVA solution for preparing the required Mg-AI-LDH-PVA spinnable solution. For homogenizing, the resulting solution was sonicated for a duration of 1 h at room temperature.

### 2.4. Electrospinning of Membrane

**Figure 1:**
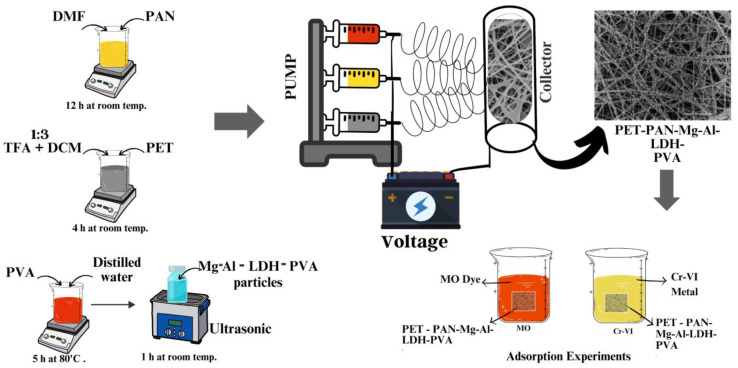
Schematic diagram of electrospinning setup with adsorption experiment.

To synthesis the PET-PAN (PP) and PET-PAN-Mg-Al-LDH-PVA (PPLH) nanocomposite membranes, the electrospinning technique was employed. Table 1 provides the details of the composition of the polymer solutions. Syringes having a capacity of 10 mL and 0.5 mm nozzle diameter were fixed onto the syringe holders and the prepared solutions were poured into them, as shown in Figure 1. For preparing the required membranes (i.e., PP, PPLH_1_, PPLH_2_, and PPLH_3_), electrospinning of the prepared solutions was carried out at a constant flow rate of 0.5 mL/h, drum speed of 30 rpm, voltage of 19 kV, and collector to tip distance of 10 cm. During co-electrospinning, three separate syringes were used for each solution of PET, PAN, and Mg-Al-LDH-PVA. The prepared membranes were collected on a drum covered with aluminum. After collection of the membranes on aluminum foil from the collecting drum, the prepared membranes were dried at room temperature for 24 h. With the help of tweezers, the membranes were peeled off the collecting drum and subsequently used for the adsorption experiments.

### 2.5. Characterization of Electrospun Membranes

To verify the physical, mechanical, and chemical properties of the membranes, different types of techniques were used. For checking the membrane’s surface morphology, a JSM-IT 100 scanning electron microscope (SEM) (JSM-IT 100, JEOL, Tokyo, Japan) was used. The SEM analysis was conducted at an accelerating voltage of 15.0 kV. The samples to be analyzed were coated at 20 mA current for 50 sec using an auto fine coater (JEC-3000FC, JEOL, Tokyo, Japan). ImageJ 1.54d software was used to determine the mean fiber diameter distribution of the SEM images in which at least 50 fibers were measured to obtain the fiber diameter distribution. Energy dispersive X-ray spectroscopy (EDX) (JSM-IT 100, JEOL, Tokyo, Japan) was used for analysis of the chemical composition and chemical properties of the membrane surface at an accelerating voltage of 20.0 kV. Fourier-transform infrared spectroscopy (FTIR) (Spectrum two, Perkin Elmer, Waltham, MA, USA) was used for analysis of the chemical structure and functional groups present. The FTIR spectra were analyzed in the wavenumber range from 3500 to 500 cm^−1^. X-ray diffraction (XRD) (D8 Advance X-ray diffractometer, Bruker, Mannheim, Germany) was used for the determination of crystal orientation and phase structure. XRD was performed over a 2θ range from 5° to 80° for the scanning of diffraction patterns. Atomic force microscopy (AFM) (5500, Agilent technologies, Chandler, AZ, USA) was used for the analysis of the membrane’s surface roughness using the AFM height images of 3 × 3 μm areas. The water contact angle was used to check the hydrophilicity properties of the developed membranes. The tensile strength test was conducted using a universal strength tester (910 Titan 3, James Heal, Halifax, UK) for measuring the mechanical properties of the membranes at room temperature.

### 2.6. Batch Adsorption Studies

To assess the membrane’s adsorption properties, batch adsorption experiments were conducted. A 50 mL beaker containing 20 mL of Cr(IV) and MO solutions was used for the batch experiments. The batch experiments for MO were carried out by varying the parameters including contact time (15, 30, 45, 60, and 90 min), pH (2.0, 4.0, 6.0, 8.0, and 10.0), membrane dosage (5.0, 15.0, 25.0, 35.0, and 45.0 mg), and MO concentration (5.0, 10.0, 15.0, 25.0, and 30.0 mg/L). Similarly, batch experiments for Cr(IV) (concentration 5 mg/L) were carried out at the optimum parameters, including contact time (90 min), pH (2.0), and membrane dosage (25.0 mg). To adjust the pH of pollutants, 0.01 M HCl and 0.01 M NaOH solutions were used. After completion of the batch experiments, the membranes were removed from the beakers. A UV-Vis dual beam spectrophotometer (L7, BioBase, Jinan, China) at a wavelength of 464 nm was used for the determination of the concentration of the MO solution. Meanwhile, the quantification of the concentration of Cr(IV) at 540 nm was carried out using the 1,5-diphenyl carbazide (DPC)-based standard method [37,38]. All experiments were performed in triplicate.

For the calculation of removal efficiency and adsorption capacity of MO and Cr(IV), Equations (1) and (2) were used [39,40]:

(1) $$q_{e=}\frac{\left(C_{o}-C_{f}\right)}{m}\times V$$

(2) $$Removal(\%)=\frac{\left(C_{o}-C_{f}\right)}{C_{o}}\times 100$$

where $“C_{o}”(mg/L)$ represents the initial concentration of MO and Cr(IV), while ${“C}_{f}”(mg/L)$ represents the final concentration of MO and Cr(IV). “*q_e_*” is used to denote the amount in mg/g of MO and Cr(VI) adsorbed on the membrane, “*m*” is the mass (g) of the membrane dosage used, and *V* is the volume of the solution in mL.

### 2.7. Reusability Experiment

One of the most important properties of membranes with respect to their practical applications and economic aspect is their ability to be reused. To assess the PPLH_3_ membrane’s stability, a reusability test was carried out for MO removal. The adsorbed PPLH_3_ membrane was kept in proportion to the amount of 0.1 M NaOH solution, then transferred into a shaker for a duration of 6 h at room temperature. In a highly alkaline medium, the adsorbed MO was discharged into the solution. The membrane was then washed with distilled water and dried at 60 °C in a vacuum dryer. The reusability experiment was repeated for five cycles.

### 2.8. Adsorption Isotherms

Langmuir and Freundlich isotherm models were used to study the adsorption isotherm. As shown by the Langmuir isotherm model, monolayer adsorption determines the maximum adsorption level. The maximum adsorption is indicated by the surface saturation point. The following Equation (3), derived from the Langmuir isotherm model, can be expressed as [41]:

(3) $$\frac{C_{e}}{q_{e}}=\frac{C_{e}}{q_{m}}+\frac{1}{{K_{L}q}_{m}}$$

where $“C_{e}”$ represents the equilibrium pollutant concentration in mg/L, “*q_e_*” is the equilibrium adsorption capacity in mg/g, “*K_L_*” is the solution affinity, while “*q_m_*” is the pollutant maximum adsorption.

The Freundlich isotherm model illustrates the multilayer adsorption process in a heterogeneous system. Equation (4), of the linear Freundlich isotherm model, can be written as [42]:

(4) $$\ln q_{e}=\ln{(k}_{f})+\ln\frac{C_{e}}{n}$$

where “1/n” represents the intensity of adsorption, while “*kf*” is a constant used in this model.

### 2.9. Adsorption Kinetics

The adsorption kinetics help to measure the rate of the adsorption process by the adsorbent. To understand the adsorption mechanism of the membrane for the solute, pseudo-1st order and pseudo-2nd order kinetics adsorption models can be used. The pseudo-1st order adsorption model shows physical adsorption, while the pseudo-2nd order kinetics model applies to chemical adsorption [43]. For the kinetics calculations, the following Equations (5) and (6) were used for the pseudo-1st order and pseudo-2nd order models, respectively [44].

(5) $$log(q_{e}-q_{t})=Logq-\frac{k_{1}}{2.303}t$$

(6) $$\frac{t}{q_{t}}=\frac{1}{k_{2}q_{e}^{2}}+\frac{t}{q_{e}}$$

where ${“q}_{t}”$ (mg/g) at time “*t*” is the adsorption capacity, ${“q}_{e}”$ is the adsorbate amount adsorbed at equilibrium (mg/g), and $“k_{1}”and{”k}_{2}”$ are the first and second order adsorption rate constants, respectively.

## 3. Results and Discussion

### 3.1. Characterization

#### 3.1.1. SEM Analysis

**Figure 2:**
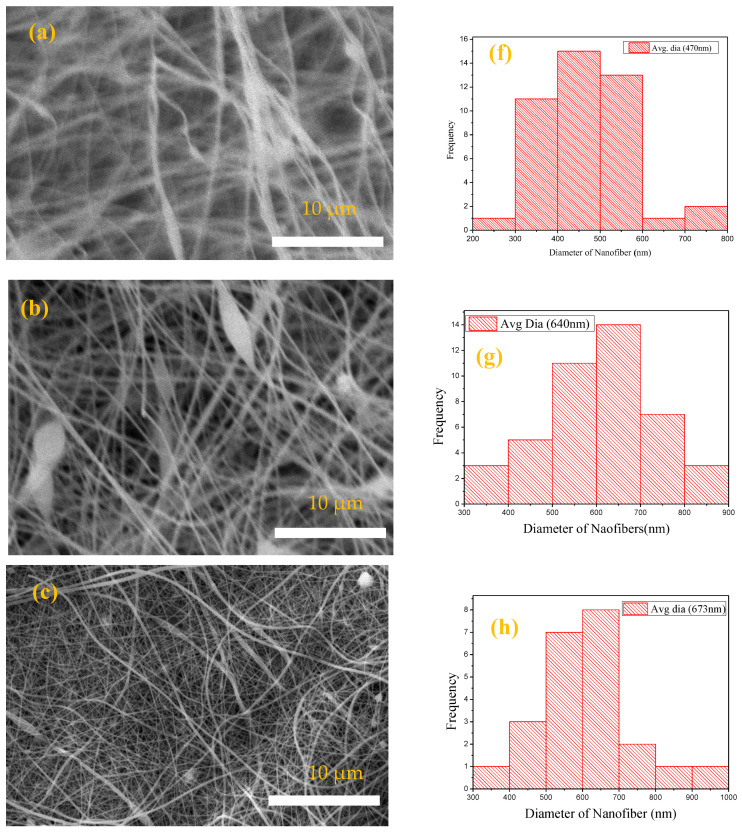

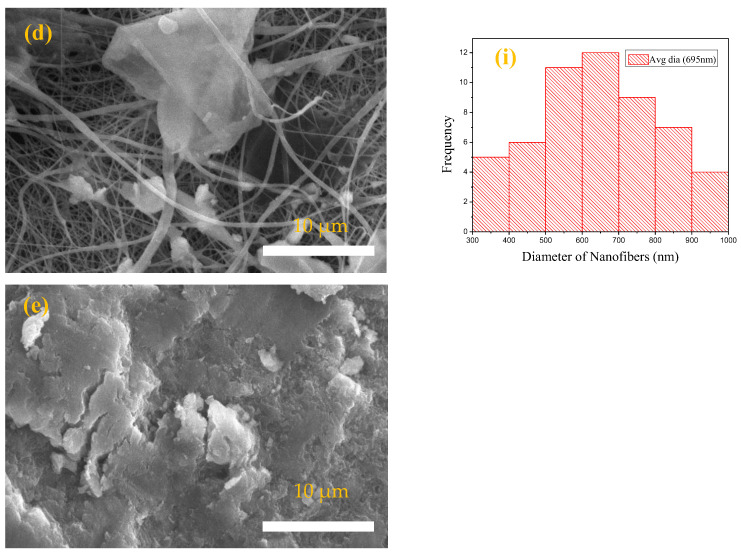
The SEM images and nanofiber diameter histograms of: (**a**,**f**) PP, (**b**,**g**) PPLH_1_, (**c**,**h**) PPLH_2_, and (**d**,**i**) PPLH_3_ membranes, and (**e**) SEM image of Mg-Al-LDH.

SEM was used for the characterization of the surface morphology of PP, PPLH_1_, PPLH_2_, and PPLH_3_ membranes having different quantities of Mg-Al-LDH nanoparticles. A bead-free morphology with smooth and uniform distribution of nanofibers was found for the PP membrane, as depicted in Figure 2a. The PPLH_1_, PPLH_2_, and PPLH_3_ membranes showed a porous and rough structure when modified with Mg-Al-LDH-PVA in contrast to the smooth structure of the PP membrane, as shown in Figure 2b–d, respectively. Mg-AI-LDH nanoparticles that were synthesized had a well-developed structure, as depicted in Figure 2e. The plate-like Mg-AI-LDH nanoparticles were found as finely dispersed porous agglomerates having irregular shapes with an average size of 0.5–1.5 µm [34,45]. ImageJ 1.54d software was used to measure the fiber diameter of the membranes from SEM images [46], as shown in Figure 2f–i. As compared to the PP (470 nm) membrane, the average diameters of the PPLH_1_ (640 nm), PPLH_2_ (673 nm), and PPLH_3_ (695 nm) membranes were found to be higher, and this may have been attributed to the modification with LDH. The viscosity of the solution is a major factor that may influence the diameter of the nanofiber. Previous studies indicated that the diameter of the nanofiber increases with increasing viscosity of the solution [3,47,48].

#### 3.1.2. EDX Analysis

**Figure 3:**
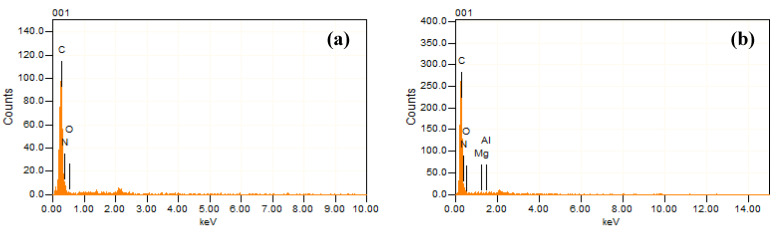

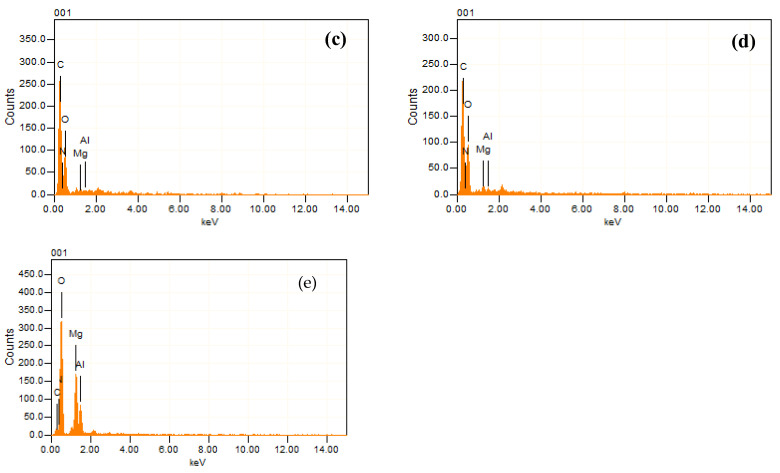
EDX spectrum of: (**a**) PP, (**b**) PPLH_1_, (**c**) PPLH_2_, (**d**) PPLH_3_, and (**e**) Mg-Al-LDH.

The EDX technique was used for determining the elemental composition of the PP, PPLH_1_, PPLH_2_, and PPLH_3_ membranes and Mg-Al-LDH nanoparticles, as shown in Figure 3a–e and Table 2. The EDX spectrum of the PP membrane exhibited carbon, oxygen, and nitrogen, as shown in Figure 3a. The elemental analysis of Mg-Al-LDH nanoparticles indicated that aluminum, carbon, magnesium, and oxygen were present, confirming the synthesis of Mg-Al-LDH nanoparticles, as illustrated in Figure 3e. The EDX analysis indicated that the PPLH_3_ membrane consisted of magnesium and aluminum at 0.94 and 0.40 atomic%, respectively. It can be observed from Table 2 that the atomic% values of elements (Mg and Al) were increased with increasing LDH dosage.

#### 3.1.3. FTIR Analysis

**Figure 4:**
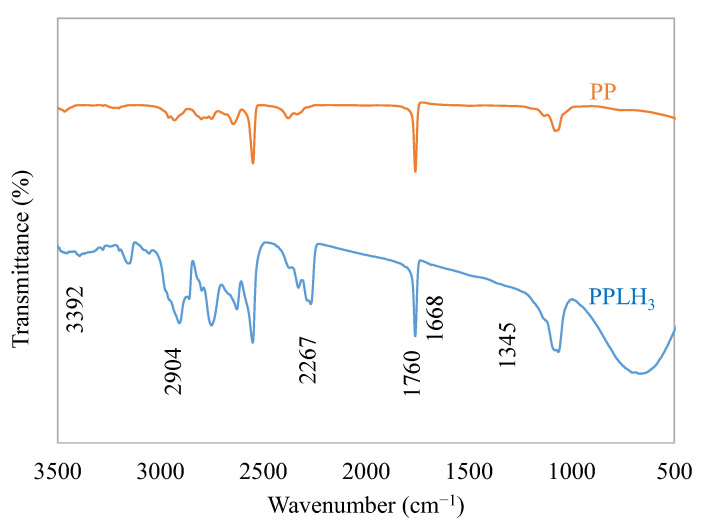
FTIR spectra for PP and PPLH_3_ membranes.

The FTIR spectra of the PP and PPLH_3_ membranes are illustrated in Figure 4. The spectrum of PAN nanofibers showed the characteristic peaks at 1452 cm^−1^ and 2267 cm^−1^ corresponding to the methylene (CH_2_) stretching vibration and nitrile group (−C≡N) stretching vibration, respectively [30]. Bands were observed for PET at 2904 cm^−1^ and 1760 cm^−1^, which may have been attributed to the methylene group (CH_2_) vibration/oscillation and longitudinal vibration of the carbonyl group (C=O), respectively [28]. In the FTIR spectrum of PVA, resonance peaks were observed at 3392 cm^−1^, caused by the presence of the –OH group, and at 2905 cm^−1^, a characteristic band indicating the presence of the C–H group [3]. The FTIR spectrum of the as-synthesized Mg-Al-LDH showed a broad band at 3392 cm^−1^ for the OH stretching vibration of hydrogen-bonded groups in the brucite-like sheets and water in the interlayer space. Peaks observed at 1345 cm^−1^ and 1668 cm^−1^ were caused by NO^3-^ intercalated in the interlayer space and the water bending vibration of interlayer water, respectively [45]. The spectra of PET, PAN, Mg-Al-LDH, and PVA exhibited characteristics bands, indicating the formation of the PPLH membrane.

#### 3.1.4. AFM Analysis

**Figure 5:**
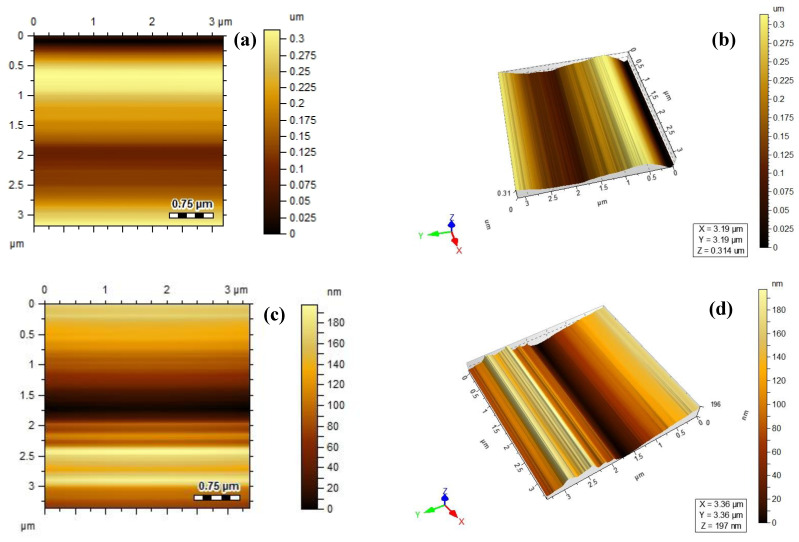
AFM results of: PP membrane (**a**) 2D and (**b**) 3D images; and PPLH_3_ membrane: (**c**) 2D and (**d**) 3D images.

The AFM test was used to characterize the surface morphology and check the surface roughness of electrospun nanocomposite membranes. Figure 5a–d displays the AFM 2D and 3D images of PP and PPLH_3_ electrospun membranes. The valley and peak values can be seen from the AFM images. The PPLH_3_ membrane exhibited an average surface roughness of 8.02 nm, while the PP membrane showed a roughness of 2.03 nm. Due to the loading of Mg-Al-LDH nanoparticles, the PPLH_3_ sample showed improved surface roughness that enhanced the adsorption and pollutant removal efficiency.

#### 3.1.5. XRD Analysis

**Figure 6:**
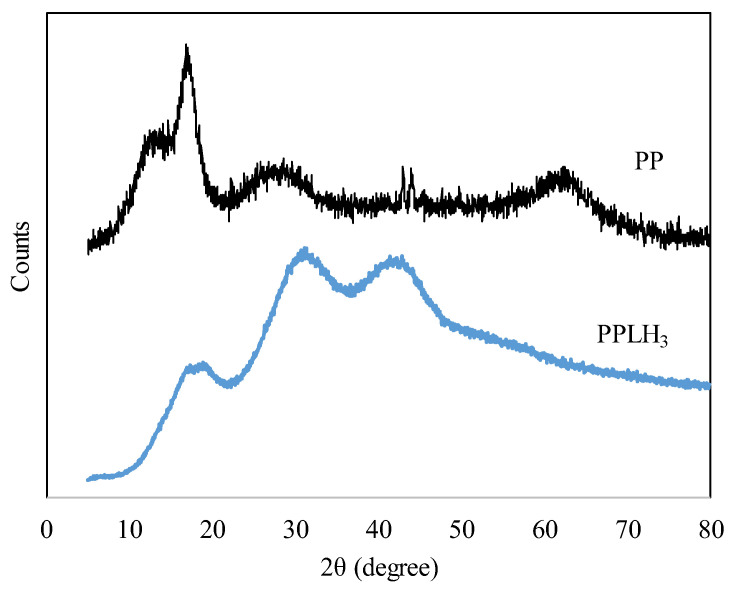
XRD patterns of PP and PPLH_3_ membranes.

The XRD spectrum analysis was conducted to check the crystal structure of the PP and PPLH_3_ membranes, as depicted in Figure 6. The diffraction peak of PET was observed at 2θ = 16° [36,49]. Meanwhile, PAN displayed the sharp diffraction peaks at 2θ = 17°, 23°, and 27° that corresponded to the crystalline structure [23,50,51]. Moreover, major Mg-Al-LDH reflections were detected at 2θ = 32° and 43° [34,52]. The PVA diffraction peak appeared at 2θ = 19° [35]. The numerous diffraction patterns indicated the fabrication of the PPLH membrane.

#### 3.1.6. Water Contact Angle Measurement

**Figure 7:**
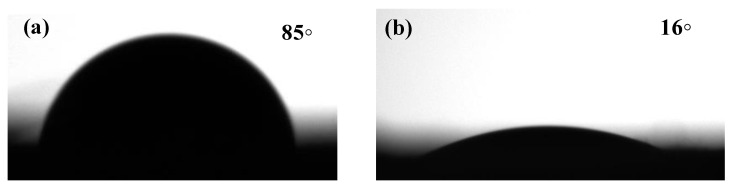
Water contact angles of (**a**) PP and (**b**) PPLH_3_ membranes.

The hydrophilicity of the PP and PPLH_3_ membranes was measured using the water contact angle. The results of the water contact angle measurements are depicted in Figure 7a,b. A decrease in the water contact angle was observed in the PPLH_3_ membrane as compared to the PP membrane [53]. The contact angle of the PP membrane was 85°, which was higher than that of the PPLH_3_ membrane at 16°. The membrane wettability shows the function of membrane hydrophilicity and surface roughness [54,55,56,57]. It was observed that the addition of Mg-Al-LDH-PVA could increase the hydrophilicity of the PPLH_3_ membrane [58,59]. It was further shown that the PPLH_3_ membrane resulted in a reduced contact angle, leading to enhanced adsorption and pollutant removal efficiency.

### 3.2. Basic Adsorption Experiment

**Figure 8:**
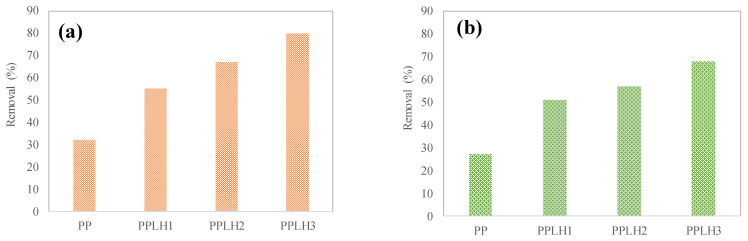
Basic adsorption experiment results for removal of (**a**) MO and (**b**) Cr(VI).

Initially, various experiments were carried out to check the removal efficiencies of the PP PPLH_1_, PPLH_2_, and PPLH_3_ membranes for Cr(VI) and MO. The adsorption experiment results are shown in Figure 8a,b. It can be observed from the basic results that the PPLH_3_ membrane had higher removal efficiencies of 80% and 68%, as compared to other membranes, for MO and Cr(VI), respectively. The higher adsorption efficiencies were due to fact that the PPLH_3_ membrane has functional groups, i.e., –OH, C–N, C–O, etc. (as shown in the FTIR results, Figure 4), that can remove pollutants through surface phenomena. Additionally, the PPLH_3_ membrane showed improved adsorption efficiencies due to better hydrophilicity, as shown in the contact angle results in Figure 7. The subsequent experiments for isotherm and kinetics models were performed using the PPLH_3_ membrane. The parameters for checking the adsorption capacities of the membrane for MO and Cr(VI) are shown in Table 3. The adsorption of Cr(VI) obtained a maximum at pH 2, as reported by several other studies [60,61].

### 3.3. Optimization of Different Parameters for MO Adsorption

#### 3.3.1. Effect of pH

**Figure 9:**
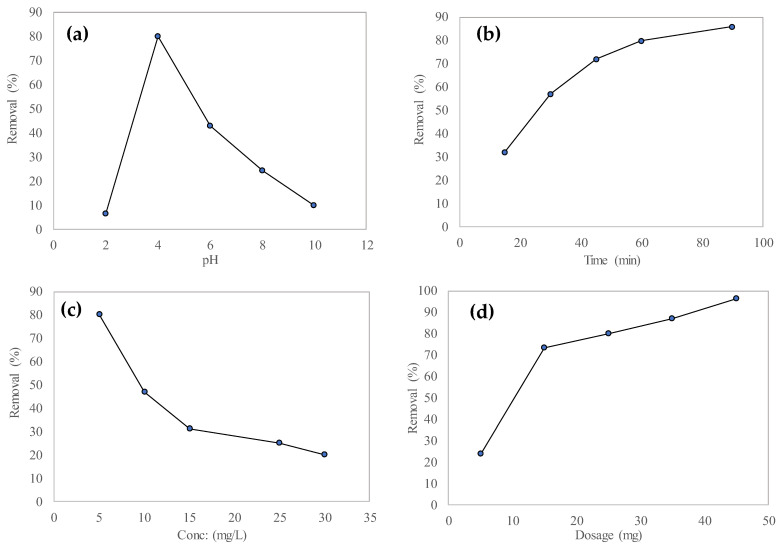
Effect of (**a**) pH, (**b**) contact time, (**c**) concentration, and (**d**) adsorbent dosage on MO adsorption using PPLH_3_ membrane.

One of the most important parameters that influences the process of adsorption is the pH of the solution. This pH value affects both the adsorption behavior and the efficiency of the adsorption process. The removal efficiency of this process can be varied by bringing about a change in the solution’s initial pH, affecting the characteristics of the solution as well as the membrane surface charge [62]. The MO adsorption experiment was carried out under conditions where the pH value of the solution was varied at 2, 4, 6, 8 and 10, while constant values of the initial dye concentration (5 mg/L), membrane dose (25 mg), and contact time (60 min) were used. At a pH value of 4, the MO adsorption showed a maximum removal efficiency of 80%, as given in Figure 9a. From the results of the experiments, it was found that the removal efficiency of MO showed decreasing values with an increase in the pH of the solution. At low values of pH (4), the higher adsorption efficiency could be attributed to the adsorbent’s protonation properties [30,44]. As it was observed that greater MO removal was obtained at lower pH values, further adsorption experiments were conducted at a pH value of 4.

#### 3.3.2. Effect of Contact Time

Several experiments were conducted to determine the optimum equilibrium time that tends to provide the maximum removal of MO. At different contact times (15, 30, 45, 60, and 90 min) the adsorption efficiency values for MO are shown in Figure 9b. An increase in the adsorption efficiency values for MO were obtained at the initial contact time due to the availability of a higher number of adsorption active sites on the surface of the PPLH_3_ membrane. A slow trend in the adsorption efficiency was observed until the equilibrium condition was reached at 60 min duration. It has been noted that each polymer has a different equilibrium time because of the availability of active sites on the membrane surface and a decrease in the abundance of active functional groups on the surface of the adsorbent [63,64,65]. Therefore, it could be concluded that the appropriate time for the adsorption process was 60 min.

#### 3.3.3. Effect of Initial Dye Concentration

To analyze the effect of initial dye concentration on the adsorption of MO, the initial dye concentration was varied between 5 and 30 mg/L. A decrease in the adsorption efficiency of MO was observed with an increase in initial dye concentration, and the maximum adsorption efficiency was obtained at 5 mg/L, as illustrated in Figure 9c. At the same adsorption times, an increase in the initial dye concentration resulted in a decreased percentage of MO removal [40,66].

#### 3.3.4. Effect of Adsorbent Dosage

Another important parameter that influences a material’s adsorption performance is the dosage of the adsorbent used. The removal efficiency of MO was analyzed by varying the adsorbent dosages of PPLH_3_ in the range of 5~45 mg. It was observed that increased dye uptake by the membrane was obtained with an increase in adsorbent dosage [67]. The increase in the number of active sites on the surface of PPLH_3_ caused by the corresponding increased dosage of the adsorbent enhanced the adsorption process [68]. From the results of these experiments, it could be concluded that a PPLH_3_ dosage of 45 mg provided the maximum 96.2% MO removal, as shown in Figure 9d. The optimum PPLH_3_ dosage for further experiments was accordingly decided as 25 mg.

### 3.4. Adsorption Isotherms

**Figure 10:**
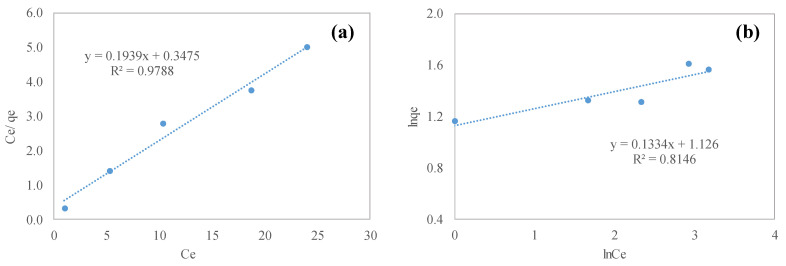

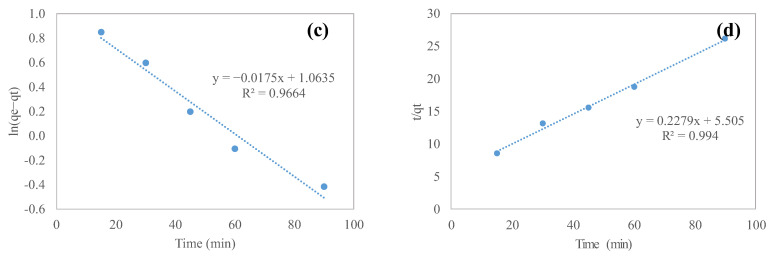
The (**a**) Langmuir and (**b**) Freundlich isotherm models for MO adsorption. (**c**) pseudo-1st order and (**d**) pseudo-2nd order kinetic curves of MO adsorption.

To investigate the adsorption isotherm behavior of the PPLH_3_ membrane, Langmuir and Freundlich models were used. In order to designate the functions of the adsorbate amount binding to the surface of the adsorbent, an adsorption isotherm study was carried out [69]. A comparison of the parameters obtained from the Langmuir and Freundlich isotherms for the adsorption of MO on the PPLH_3_ membrane is illustrated in Figure 10a,b, respectively, and Table 4. For MO adsorption on PPLH_3_, the Langmuir isotherm was found ideally suited with an R^2^ value of 0.978. In both isotherms, a linear-shaped graph was obtained. The Langmuir isotherm was found to be adequately applied to the adsorption process and ideally suited to PPLH_3_, having a maximum MO adsorption capacity of 5.2 mg/g, as shown in Table 4.

### 3.5. Adsorption Kinetics

The pseudo-1st order and pseudo-2nd order kinetic parameters were determined and the R^2^ correlation coefficients were obtained as 0.966 and 0.994, respectively, as shown in Table 5 and Figure 10c,d. From the analysis of the correlation coefficients and experimental data, it was noted that, as compared to pseudo-1st order kinetics, pseudo-2nd order kinetics provided a better illustration of the MO adsorption process, as shown in Figure 10d. It could be concluded that pseudo-2nd order kinetics model was the best fit for this study, which inferred that the chemisorption mechanism was followed during the adsorption process [70,71].

### 3.6. Mechanical Behavior

**Figure 11:**
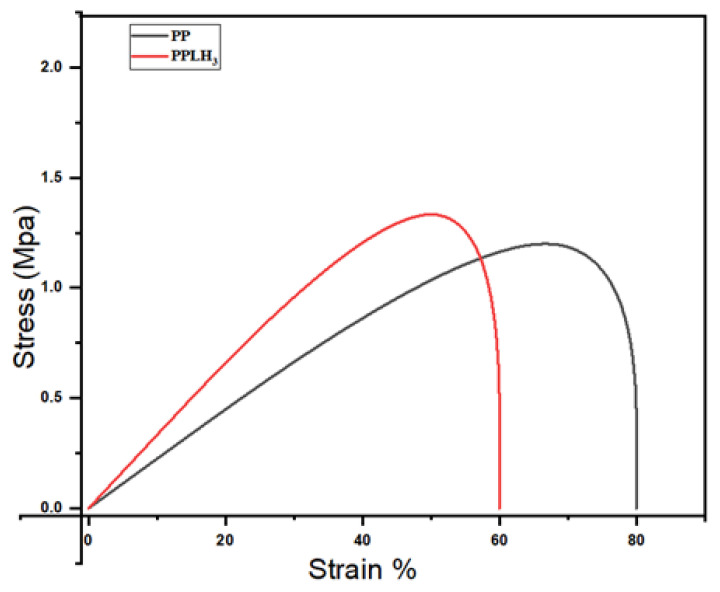
Stress–strain curve of PP and PPLH_3_ membranes.

The mechanical properties of the PP and PPLH_3_ nanofiber membranes were measured by tensile tests, and the stress–strain curves of samples are shown in Figure 11. The results displayed that the tensile strength of the PPLH_3_ membrane was higher than the PP membrane, having values of 1.4 and 1.2 MPa, respectively. This may have been due to the change in crystalline behavior achieved during the modification of the PP membrane with Mg-Al-LDH-PVA (as shown in the XRD results, Figure 6). The increase in yield stress shows an improvement in the membrane resistance to deformation under the condition of tensile stress [58]. The electrospun membrane’s mechanical properties mainly depend on the fiber structures and fiber interactions [72]. It was observed from the literature that larger fiber diameters of electrospun membranes can improve their mechanical properties [58,73].

### 3.7. Recyclability Test

**Figure 12:**
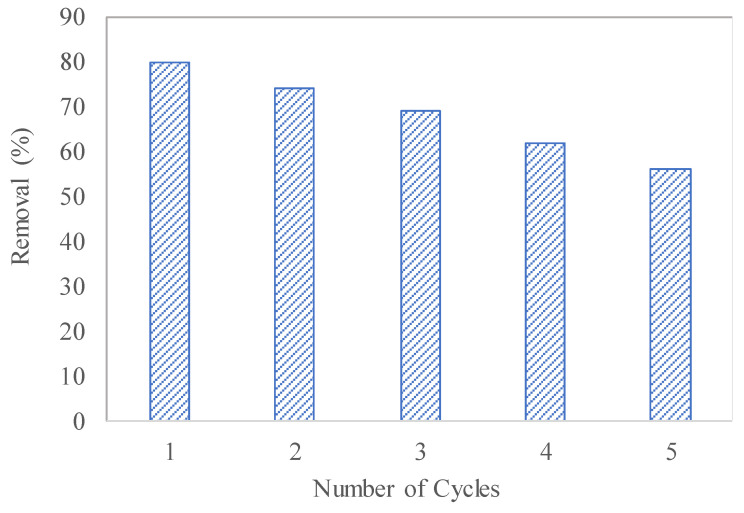
Recyclability test of PPLH_3_ membrane for MO adsorption.

Five cycles of adsorption experiments for MO were conducted to test the stability of the PPLH_3_ membrane. On the first run, the MO removal rate was 80%, which decreased for the subsequent number of cycles. Lastly, the removal rate was 56% for MO after the fifth run, as shown in Figure 12. The PPLH_3_ membrane showed good adsorption performance for up to five cycles. This meant that the developed PPLH_3_ membrane had good stability due to the modification with Mg-Al-LDH-PVA, which could be verified from the results of the FTIR and tensile strength analyses, as shown in Figure 4 and Figure 11, respectively. The PPLH_3_ membrane performance compared with that of related materials reported in other research studies is shown in Table 6. It can be observed from the results that the PPLH_3_ membrane could efficiently remove pollutants as compared to other materials.

### 3.8. Proposed Adsorption Mechanism

**Figure 13:**
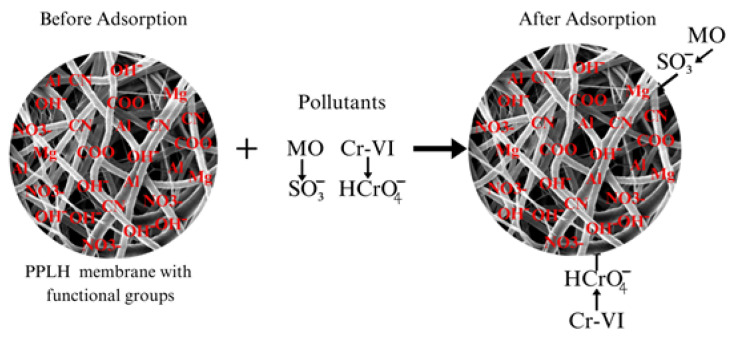
Proposed adsorption mechanism of MO and Cr(VI) using PPLH_3_ membrane.

The process of adsorption is mainly because of surface phenomena and it is used for the capturing of pollutants between two mediums, such as at the solid–liquid interface. The process of adsorption occurs due to the force of attraction between the adsorbate and adsorbent [76,77]. Therefore, the proposed mechanism of adsorption between the PPLH_3_ membrane, MO, and Cr(VI) is illustrated in Figure 13. The PPLH_3_ membrane contains different functional groups, as shown in the FTIR results (Figure 4). The MO molecules, having the SO_3_^−^ functional group, are adsorbed on the surface of PPLH_3_ because of electrostatic attraction, hydrogen bonding, and surface complexation [78,79,80,81]. Similarly, adsorption of Cr(VI) occurs due to the likely participation of hydroxyl ions on the surface of the PPLH_3_ membrane through electrostatic attraction and anion exchange [82].

## 4. Conclusions

In this study, the fabrication of a novel PET-PAN embedded with Mg-AI-LDH hybrid nanocomposite membrane was successfully achieved via the electrospinning process and verified by SEM, EDX, FTIR, XRD, and AFM analyses. Improved hydrophilicity and tensile property of the PPLH_3_ membrane were observed after adding Mg-Al-LDH-PVA. The adsorption results demonstrated that the PPLH_3_ membrane showed significant adsorption efficiencies of 68% and 80% for Cr(VI) and MO, respectively. The adsorption of Cr(VI) and MO was pH dependent. From the recyclability experiments, it was observed that the adsorption efficiency of the PPLH_3_ membrane was stable for five cycles. The adsorption characteristics of the PPLH_3_ membrane obeyed the Langmuir isotherm model and pseudo-2nd order kinetics model with R^2^ values of 0.97 and 0.99, respectively. As a result, the PPLH_3_ membrane may be used as a potential adsorbent because of its excellent adsorptive properties and recyclability for various other pollutants in wastewater in the future.

## Figures and Tables

**Table 1:** Electrospinning polymer solutions for synthesis of PP and PPLH membranes.

Sr #	Membrane Type	PET (%)	PAN (%)	LDH (g)	PVA (%)
1	PP	5	8	_	_
2	PPLH_1_	5	8	0.08	8
3	PPLH_2_	5	8	0.12	8
4	PPLH_3_	5	8	0.16	8

**Table 2:** EDX atomic composition of PP, PPLH_1_, PPLH_2_, PPLH_3_, and Mg-Al-LDH.

Sample	Al	Mg	C	O	N
LDH	5.39	16.63	11.37	66.61	-
PP	-	-	86.61	6.95	6.43
PPLH_1_	0.17	0.20	72.71	23.11	3.81
PPLH_2_	0.22	0.42	58.17	36.10	5.09
PPLH_3_	0.40	0.94	57.29	37.34	4.03

**Table 3:** Basic adsorption experiments for MO and Cr(VI) using PP and PPLH membranes.

Membrane Type	Pollutant Type	Solution pH	Adsorption Time (min)	Concentration (mg/L)	Dosage (mg)	Solution Volume (mL)
PP	Cr(VI)	2	90	5	25	20
PPLH_1_						
PPLH_2_						
PPLH_3_						
PP	MO	4	60	5	25	20
PPLH_1_						
PPLH_2_						
PPLH_3_						

**Table 4:** The Langmuir and Freundlich isotherm model parameters for MO adsorption.

Langmuir Isotherm			Freundlich Isotherm		
q_max_ (mg g^–1^)	KL (L mg^–1^)	R^2^	KF (mg·g^−1^)(L·mg^−1^)1/n	1/n	R^2^
5.2	0.558	0.9788	3.1	0.1	0.8146

**Table 5:** The kinetic parameters of pseudo-1st order and pseudo-2nd order for MO adsorption.

Pseudo-1st Order			Pseudo-2nd Order		
q_e_ (mg g^–1^)	k_1_ (h^–1^)	R^2^	q_e_ (mg g^–1^)	k_2_ (g mg^–1^ h^–1^)	R^2^
2.90	0.018	0.966	4.39	0.009	0.994

**Table 6:** Comparison of this study with other related studies for pollutant adsorption.

S.No	Adsorbent	pH	Pollutant	Equilibrium Time (min)	q_max_ (mg/g)	Reference
1	PET-PAN-Mg-AI-LDH-PVA	4	MO	60	5.2	This study
2	NiFeLDH/PAN/GO	6	Cr(VI)	-	6.19	[31]
3	Fe NPs/SiO_2_–NH_2_/glycerol	3	MO	180	3.02	[74]
4	LDH@Fe_3_O_4_/PVA	6	MO	420	19.5	[75]
5	Zein/nylon-6 nanofibrous membrane	2	Cr(VI)	60	4.73	[65]
6	Chitosan/nylon 6	3	Cr(VI)	240	23.9	[61]

## Data Availability

Data are included in the manuscript.

## Supplementary Materials

The following supporting information can be downloaded at: https://www.mdpi.com/article/10.3390/polym15224388/s1, Figure S1: Schematic diagram of synthesis of Mg-Al-LDH nanoparticles.
