# Supplementary material for: Development of Novel PET-PAN Electrospun Nanocomposite Membrane Embedded with Layered Double Hydroxides Hybrid for Efficient Wastewater Treatment
Source: Polymers (Basel). 2023 Nov 12;15(22):4388. doi: 10.3390/polym15224388 (PMC10674731; doi:10.3390/polym15224388)
Supplement: Supplementary file 1 [file polymers-15-04388-s001.zip › polymers-2694426-supplementary.pdf]

polymers
MDPI

Supplementary data

Type of the Paper (Article)

Development of novel PET-PAN electrospun nanocomposite membrane embedded with Layered double hydroxides-hybrid for efficient wastewater treatment

Abdul Majeed Pirzada [1,*], Imran Ali [1,*], Nabi Bakhsh Mallah [2] and Ghulamullah Maitlo [3]

1 Department of Environmental Sciences, Sindh Madressatul Islam University, Karachi, Pakistan; ampirzada@smiu.edu.pk (A.M.P); imranali@hanyang.ac.kr ; imran.ali@smiu.edu.pk (I.A)
2 Faculty of Engineering, Science and Technology, Hamdard University, Karachi, Pakistan; nabi.bakhsh@hamdard.edu.pk
3 Department of Chemical Engineering, Dawood University of Engineering and Technology, Karachi, Pakistan; ghulam.maitlo@duet.edu.pk
* Correspondence: ampirzada@smiu.edu.pk (A.M.P); imranali@hanyang.ac.kr (I.A)

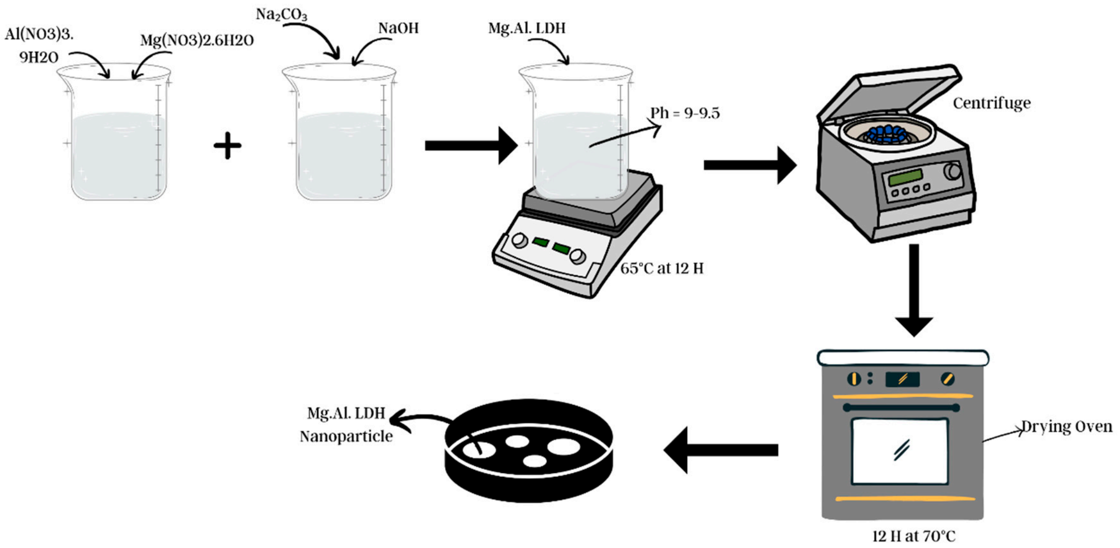

Figure S1. Schematic diagram of synthesis of Mg-Al-LDH nanoparticles.
